# Use of complementary and alternative medicine (CAM) among people living with Sjögren’s: a cross-sectional survey using a modified international CAM questionnaire (I-CAM-Q)

**DOI:** 10.1007/s00296-025-05802-w

**Published:** 2025-02-20

**Authors:** Michelle Flood, Joan Ní Gabhann-Dromgoole, Gráinne Tynan, Niamh Dillon, Deirdre Collins, Monika Lauder, Eileen Sheehy, Frank Moriarty, James W. Barlow

**Affiliations:** 1 School of Pharmacy & Biomolecular Sciences, RCSI University of Medicine and Health Sciences, Dublin, Ireland; 2 RCSI PPI Ignite Network, Office of Research and Innovation, RCSI University of Medicine and Health Sciences, Dublin, Ireland; 3 Sjögren’s Ireland Advocacy Group, Dublin, Ireland; 4 The SYNERG-IE Programme, School of Pharmacy & Biomolecular Sciences, RCSI University of Medicine and Health Sciences, Dublin, Ireland; 5 Department of Chemistry, RCSI University of Medicine and Health Sciences, Dublin, Ireland

**Keywords:** Therapies, Complementary, Medicine, Integrative, Sjögren’s syndrome, Surveys, Questionnaires

## Abstract

Sjögren’s is a chronic autoimmune disease with diverse symptomatology, and varying patient satisfaction with management. Patients with chronic conditions are known to be higher users of complementary and alternative (CAM) practices, yet little information regarding extent of such use exists. This paper describes usage of CAM practices among people living with Sjögren’s, encompassing visits to healthcare providers, CAM practitioners, self-administered CAM and self-help practices. We explored both purposes and perceived helpfulness of the various modalities utilised. 296 respondents completed an online survey administered through Sjögren’s Research Ireland in 2023. An adapted form of the International Complementary and Alternative Medicine Questionnaire (I-CAM-Q) was utilised as the survey instrument. 88.5% of respondents had a formal diagnosis of Sjögren’s. The majority (93.6%) were female, across all age groups. Over half (52%) of non-retired respondents were at least partially unable to work due to their diagnosis. Over half of all respondents (58.8%) listed at least one concomitant health problem. Most respondents (248, 83.8%) had used some form of CAM within the preceding 12 months. One-in-four had attended a CAM practitioner, most commonly chiropractors (8.4%) or acupuncturists (7.8%). Conventional healthcare and CAM providers were both consulted more commonly for management of chronic conditions or for well-being than for acute symptom management, although this was particularly stark for CAM consultations. 196 respondents (66.2%) reported use of self-administered CAM, predominantly vitamins/minerals or dietary supplements. 69.9% used various self-care practices, with the most reported being meditation, relaxation techniques and prayer. People living with Sjögren’s attend both conventional healthcare providers and CAM practitioners to a high degree and use a diverse range of complementary therapies and practices. Health care professionals need to consider and discuss potential CAM use by this patient cohort and offer evidence-based patient education about therapies and practices encountered.

**Supplementary Information:**

The online version contains supplementary material available at 10.1007/s00296-025-05802-w.

## Introduction

Sjögren’s is a chronic autoimmune disease of unknown aetiology, classically characterised by dryness of the eyes, mouth and other mucosal surfaces. In addition, systemic manifestations are experienced by over 70% of patients, and include fatigue, arthralgia and multi-organ involvement [1]. The impact of Sjögren’s on diverse aspects of daily living and functioning has been recently highlighted, and the need to thus factor quality of life assessments into all aspects of research, planning and care [2]. Treatment paradigms for Sjögren’s have traditionally focused on symptom relief or immune suppression, although the diversity of symptomatology among patients is increasingly recognised and requires an emphasis on personalised treatment plans [3].

Many factors influence the satisfaction of people living with Sjögren’s with the management of their condition, however recent evidence points to a significant proportion not being fully satisfied [4]. Dissatisfaction among those living with chronic disease such as Sjögren’s is often cited as a reason why this broader patient demographic may consider engagement with some form of complementary and alternative medicine (CAM). CAM is described by the World Health Organisation (WHO) as a broad set of health care practices that are not part of that country’s own tradition or conventional medicine and are not fully integrated into the dominant healthcare system [5]. In a systematic review of factors that influence usage of CAM worldwide, dissatisfaction with conventional medicine was one of three primary reasons that emerged, alongside an expectation of benefit and perceived safety [6]. Although some CAM interventions have shown promise, many variables must be considered in their optimal use, while others lack any evidence-base. For the most part, across jurisdictions there remains a lack of appropriate regulation of CAM practitioners.

There have been very few publications in the literature concerning CAM use in people living with Sjögren’s. The use of CAM by people living with Sjögren’s was first noted by Pal in a 1998 letter [7], yet since then, few additional reports on the extent of CAM use in this population have emerged. Lu et al. explored the associations of clinical manifestations of Systemic Lupus Erythematosus (SLE; based on the definition of the SLE Disease Activity Index 2000 and their frequency of occurrence) with CAM use in 317 female Taiwanese patients [8]. It was noted that different manifestations were associated with the use of different CAM therapies. Sjögren’s was the third most prevalent (28.4%) manifestation reported but was not significantly associated with the use of any of 10 named complementary therapies. Among 121 patients with SLE, primary Sjögren’s, or systemic sclerosis surveyed at a French university hospital, 55% utilised CAM. Of the 186 CAM interventions recorded, the most common were osteopathy, homeopathy, and acupuncture. Motivations for CAM use included improvement in well-being (22%), reducing fatigue (18%) and pain (33%). Subjective improvement in mental well-being was reported in 89% of users. CAM use was associated with Western culture, being professionally active, and having poor quality of life and higher anxiety scores [9].

Due to the complexity of Sjögren’s, and possible treatment for co-morbidities, it is important to document all therapeutic interventions utilised, which can inform perspectives on their condition(s), self-management strategies, and the potential for drug-drug interactions and toxicities. This includes the use of CAM practices, which may remain undisclosed unless specifically discussed with people living with Sjögren’s. The international CAM questionnaire (I-CAM-Q) is a tool developed to methodically evaluate CAM use in diverse patient populations and allow cross-study comparisons. It was designed to elicit frequency, purpose and satisfaction with various practices [10]. It allows data collection concerning visits to health care providers, complementary treatments from practitioners, use of herbal medicines and dietary supplements, and self-help practices. The I-CAM-Q has been used in diverse contexts [11–13], translated into various languages, and applied in abridged and modified versions. It has been utilised in general populations and specific patient cohorts, including in chronic diseases such as multiple sclerosis (MS) [14], although, to date, not in Sjögren’s. The lack of available data on CAM use in the Sjögren’s community, coupled with its identification as a priority area for Sjögren’s Research Ireland were the stimuli for this work. Our aim in the study was thus to establish the extent of CAM use among a cohort of people living with Sjögren’s, and to investigate their motivations in doing so.

## Methods

This cross-sectional study was conducted as a collaborative project between academic researchers and Sjögren’s Ireland, an advocacy group for those living with Sjögren’s in Ireland. It was entirely administered via the online SurveyMonkey^®^ platform, between Monday 16th October 2023 and Monday 13th November 2023.

### Study population

Recruitment for the online survey was carried out at the annual online Sjögren’s Ireland webinar event and via the social media of Sjögren’s Ireland and RCSI researchers. The inclusion criteria were individuals living with Sjögren’s or those with signs and symptoms of Sjögren’s awaiting formal diagnosis. This is because it is recognised that many people experience delays of up to 10 years in obtaining a formal diagnosis.

### Ethics

The project was approved by the RCSI Research and Ethics committee (REC 202105006). The study adhered to the tenets of the Declaration of Helsinki. Informed consent was obtained from all participants prior to participation via a series of consent questions on the front page of the survey, with all participants supplied with information about the aims and objectives of the study prior to participation. No identifying information was collected and so the survey data was considered to be anonymous. We observed all salient points on designing and reporting survey studies to reflect best practice in the literature [15].

### Research instrument

A survey instrument with both open- and closed-ended questions, including Likert scale questions, was developed by Sjögren’s Ireland Public Patient Involvement (PPI) contributors and RCSI researchers, many of whom were also healthcare professionals (Supplementary Table 1). Questionnaire domains included demographic information (7 questions), with detailed questions on CAM adapted from the previously reported and validated questionnaire known as I-CAM-Q. This comprised four sections: (a) visits to health providers (3 questions), (b) complementary treatments via conventional practitioners (4 questions), (c) use of herbal medicine and dietary supplements (3 questions), and (d) self-help practices (4 questions). Additionally, we sought information on adverse events (2 questions). The survey instrument allowed partial completion, including skipping of questions. PPI contributors reviewed the surveys in relation to accessibility and inclusion, relevance, necessity, language, and overall questionnaire length. Adjustments were made in response to their feedback and included recommendations to provide a pause option to reduce the amount of screen time required, due to common Sjögren’s symptoms of dry eye, fatigue and brain fog.

### Statistical analysis

The characteristics of the survey respondents were described. We summarised the number of respondents reporting attendance at each type of healthcare provider, receipt of CAM treatments from providers, and use of self-administered CAM treatments and self-help practices. For each, the principal reason cited for attendance/treatment (acute illness, chronic illness, or well-being), and the perceived helpfulness (not, somewhat, or very helpful) were summarised. Percentages for each question are reported based on all respondents (frequency of use) or all respondents for whom the question is relevant (reason and helpfulness), i.e. including non-responders to each question. Subgroup analysis was conducted to assess whether frequency of use, reason, and perceived helpfulness differed based on respondent location (Ireland versus elsewhere) and age group (aged < 60 years or ≥ 60 years). Differences were assessed using chi squared tests or Fisher’s exact tests where there were fewer than 5 respondents in a category, and statistical significance was defined as *p* < 0.05. Analysis was conducted in Stata v.18 (College Station, TX: StataCorp LLC).

## Results

### Demographics

A total of 296 respondents completed the survey. Of those, 262 (88.5%) had a formal diagnosis of Sjögren’s while 27 (9.1%) had symptoms of Sjögren’s without a formal diagnosis. Table 1 presents the demographic characteristics of the survey population. Most respondents (277, 93.6%) were female, with respondents broadly distributed across all age groups. Over half (164, 55%) of the respondents were located in Ireland, with most of the remainder from Europe or North America. A sizeable proportion (52%) of non-retired respondents were either not working or working reduced hours due to their diagnosis. With respect to concomitant conditions, over 100 concomitant conditions were listed, with 174 respondents (58.8%) naming at least one such health problem, with the most frequent conditions reported being fibromyalgia, lupus and rheumatoid arthritis. Upon aggregation of the results for the four dimensions of the ICAM-Q, 248 (83.8%) of the respondents had used some form of CAM within the last 12 months; the results for each aspect are now described.

**Table 1:** Demographics of the survey population

Characteristic	Category	Number
Sex	Male	15
	Female	277
	Non-binary	0
	Other	0
	Prefer not to say	1
Age range	18–29	4
	30–39	33
	40–49	56
	50–59	84
	60–69	71
	70–79	39
	80–89	6
	Other	0
County of origin	Ireland	164
	UK	56
	Mainland Europe	18
	North America	43
	Other	13
Occupation	Working full-time	85
	Working reduced hours due to disability	40
	Unable to work due to disability	52
	Retired	75
	Made redundant	3
	Other	37
Common concomitant medical conditions	Fibromyalgia	26
	Lupus (systemic & cutaneous)	24
	Rheumatoid arthritis	24
	Osteoarthritis	22
	Hypothyroidism	20
	Raynaud’s	19
	Neuropathy	19
	Coeliac disease	12
	Hashimoto’s thyroiditis	10
	Dysautonomia/POTS	8

### Attendance at healthcare providers

A majority (263, 88.9%) of respondents had attended their general practitioner (or other doctor) in the past year, while 104 (35.1%) had attended a physiotherapist. One in four (74, 25%) respondents had visited at least one CAM practitioner during the past year. Overall, the more commonly visited CAM practitioners (Table 2) were chiropractors (8.4%) and acupuncturists (7.8%). The ‘other’ category included conventional practitioners, such as specialist doctors, primarily rheumatologists (24, 8.1%) or dentists (13, 4.4%), although 21 (7.1%) of this category were diverse CAM practitioners. Principal reasons cited in visiting CAM practitioners were to treat a long-term (> 1 month) health condition or its symptoms, or to improve wellbeing. In all cases, a majority of patients found attendance at the healthcare providers either very or somewhat helpful. In most cases, there was no difference between respondent subgroups (Supplementary Table 2). There was a higher proportion reporting chiropractor use outside of Ireland (12.9% vs. 4.9%, *p* = 0.014), and a higher proportion reporting physiotherapist use among those aged ≥ 60 years (42.0% vs. 30.5%, *p* = 0.042). More of the respondents aged ≥ 60 years rated their doctor as helpful compared to younger respondents (94.3% vs. 84.7%, *p* = 0.016).

**Table 2:** Attendance at health care providers: frequency, motivation and perceived helpfulness

Health care provider	Frequency	Acute illness	Chronic illness	Well-being	Helpfulness % (very/some)
Doctor (GP or other)	263	61	165	25	89.3
Physiotherapist	104	26	62	13	90.4
Chiropractor	25	5	14	2	90.9
Homoeopath	12	0	8	4	75
Acupuncturist	23	2	19	2	73.9
Herbalist	12	0	5	7	75
Aromatherapist	5	0	1	4	100
Spiritual healer	7	0	1	6	85.7
Other	68	7	38	17	92.3

### Complementary treatments from practitioners

A total of 102 respondents (34.4%) reported receiving a CAM treatment from a provider (Table 3). The most reported forms of CAM obtained from providers were manipulation (26.5%) and acupuncture (24.5%). Principal reasons cited for treatments were again to treat a long-term health condition (> 1 month) or its symptoms, or to improve wellbeing, with perceived helpfulness rated high for all CAM interventions. There was no difference between respondent sub-groups (Supplementary Table 3), with the exception of more individuals in Ireland versus outside reporting acupuncture being used for treating chronic illness (88.2% vs. 50%, *p* = 0.009).

**Table 3:** CAM treatments obtained from providers: frequency, motivation and perceived helpfulness

CAM treatment	Frequency	Acute illness	Chronic illness	Well-being	Helpfulness % (very/some)
Manipulation	27	3	20	4	96.3
Acupuncture	25	4	19	1	84
Herbs	13	0	8	5	84.6
Homoeopathy	12	0	11	1	75
Spiritual healing	7	0	1	6	100
Massage	6	0	0	6	83.3
Reflexology	2	0	0	2	100
Osteopathy	2	0	2	0	100
Aromatherapy	1	0	0	1	100
Other^a^	7	0	6	1	100

^a^Other comprised amatsu, reiki, peptide injections, pranic healing, sound healing, kinesiology and dry needling

### Use of herbal medicines and dietary supplements

Respondents were asked about their self-administered use of various CAM products. The results are shown in Table 4. In total, 196 individual respondents (66.2%) reported the self-administered use of herbal medicine and dietary supplements, across the four categories of CAM products. Of these, many reported the use of multiple products, and for multiple purposes, although primarily to treat a long-term health condition (> 1 month) or its symptoms, or to improve wellbeing (detail of data not shown due to complexity). In terms of individual substances, a very diverse array was reported, categorised as 52 herbal substances, 14 homoeopathic preparations, 43 dietary supplements and 20 vitamins/minerals, including multi-ingredient preparations. Overall, across all categories, the most reported were vitamin D (110 mentions), fish oil/omega acid supplements (*n* = 62) multivitamins (*n* = 42), magnesium (*n* = 34), vitamin B12 (*n* = 30), vitamin C (*n* = 27), calcium (*n* = 22) and probiotics (*n* = 22). When asked to report whether they had experienced side effects from medicines or treatments, 110 respondents (37.2%) responded affirmatively to this question, with 72.1% of this number experiencing side effects attributed to conventional medicines (Table 5), and with smaller numbers experiencing side effects from other practices. Side effects of conventional medicines were most commonly gastrointestinal (38%, including upset stomach, diarrhoea, nausea & vomiting, reflux), neurological (21%, including headache, numbness, sleepiness, twitching, migraine), immunological (21%, including rash, allergy, anaphylaxis) or miscellaneous (20%, including hair loss, pain, infection). Side effects of CAM products were almost entirely gastrointestinal in nature (nausea, stomach upset, constipation), with individual reports of pain, disease progression and weight loss.

**Table 4:** Self-selected use of CAM products

Product category	Frequency	Helpfulness % (very/some)
Herbs	46	50.7
Vitamins/Minerals	168	67.4
Homoeopathics	10	47.9
Dietary supplements	115	64.7

**Table 5:** Experience of adverse effects from treatments

Treatment	Frequency
Conventional medicine or treatment	75
Manipulation	0
Chiropractor	2
Homeopathy	1
Acupuncture	3
Aromatherapy	1
Herbs/Herbal medicine	3
Homeopathic remedies	1
Dietary supplements/Health Foods	9
Vitamins/minerals	17
Spiritual healing	0
Other (please specify)	21

### Use of self-help practices

Lastly, patients were asked about their utilisation of self-help practices in the previous 12 months (Table 6). A total of 207 responders (69.9%) indicated that they use such practices, with the most commonly encountered among users being meditation (42.5%), relaxation techniques (44.4%) and prayer (31.2%). Among the ‘other’ category, 15 diverse practices were reported, although exercise (swimming, walking, gym) (12%) and Pilates (4%) accounted for the majority of these. In the context of use, self-help practices were most used to improve wellbeing, across all practices encountered. Responses were generally consistent across subgroups (Supplementary Table 4), although a higher proportion of respondents outside Ireland reported Qigong use (6.8% vs. 1.2%, *p* = 0.014), while more of the respondents in Ireland reported Tai chi to be helpful compared to outside Ireland (87.5% vs. 16.7%, *p* = 0.026).

**Table 6:** Use of self-help practices: frequency, motivation and perceived helpfulness

CAM treatment	Frequency	Acute illness	Chronic illness	Well-being	Helpfulness % (very/some)
Meditation	88	2	22	61	91.9
Yoga	75	1	25	48	87.8
Qigong	11	0	3	7	90.9
Tai Chi	14	0	6	8	61.5
Relaxation techniques	92	2	34	53	96.7
Visualization	26	0	9	16	84.6
Aromatherapy	23	1	9	12	87
Prayer	66	0	9	53	88.9
Other	49	0	15	29	93.5

## Discussion

Across Europe, studies have shown that 25.9% of the general population had used CAM during the previous 12 months, although this varied substantially by country, ranging from 9.5 to 39.5%, with a weighted percentage of use in Ireland of 19.2% [16]. In this study, we obtained responses from 296 people, almost all of whom (88.5%) had a defined diagnosis of Sjögren’s, with the remainder symptomatic but not yet formally diagnosed. Of the respondents, 248 (83.8%) had used some form of CAM within the last 12 months. The online study attracted responses from an international cohort but was composed of a majority (55%) of Irish people, predominantly women. Demographic studies of CAM use consistently identify typical users of CAM as those with chronic health problems, particularly those more difficult to diagnose. Given that diagnostic delay has been cited as a challenge and unmet need in Sjögren’s [17], with reported times to diagnosis ranging from 2 to 6 years in some studies, this may predispose people living with Sjögren’s to utilise CAM. In our study, over 40% of patients did not receive a diagnosis for at least 5 years from symptom onset. This correlates with a recent German study investigating the elapsed time between onset of connective tissue disease symptoms and first presentation to a rheumatologist, which showed that although waiting times have diminished overall since 1980 for all connective tissue diseases, there has been no relevant improvement during the past two decades [18]. In addition, Sjögren’s is more common in women, who are also known to use CAM to a greater extent than men. The impact of Sjögren’s on ability to work is clear from our data; 52% of those of working age were either not working or working reduced hours, reflective of the burden of the condition. In the literature, work disability has been highlighted as an important modulator of Health-related quality of life (HRQoL) among people living with Sjögren’s, with reduced ability to work related to depression and fatigue demonstrated in patient cohorts across several countries [19]. This is undoubtedly exacerbated by the fact that 58.8% of respondents reported at least one co-diagnosis, the more common of these being other autoimmune conditions. Indeed, the co-existence of autoimmune diseases such as primary biliary cholangitis [20] and lupus [21] is an established phenomenon in people living with Sjögren’s, who may have one or more such linked conditions, and in the most complex cases, a multiple autoimmune disease [22].

The proportion of respondents visiting conventional practitioners in this study was very high, with 88.9% having attended their general practitioner or other doctor in the past year and 35.1% having attended a physiotherapist. Notably, I-CAM-Q does not routinely ask about attendance to physiotherapists; our results enumerate their significant contribution to the management of this patient cohort. This is further apparent when compared to the national average figure of 5.94% of people aged 50 years or more annually attending a physiotherapist [23]. This age group represents a majority of respondents in this study. A smaller yet still substantial proportion (25%) had visited at least one CAM practitioner during the same period. This contrasts somewhat with figures reported from other contexts. In a study within a multi-ethnic population of older adults in North Carolina, US, while 91.5% of respondents had attended a physician in the past year, only 7.6% had visited a CAM practitioner during the same period [24]. Conversely, in a web-based survey of the use of CAM and conventional medicine in southern Sweden, 88% had seen a conventional practitioner while 32.9% had visited a CAM provider [25]. In a more specific population of patients in Iran with MS, another chronic disease, arguably more comparable to our population than general populations, 38% of patients had visited a CAM provider in the last 12 months [14]. In our study, one in four respondents reporting GP, physiotherapist or chiropractor visits attended for acute symptom management, with two-thirds of visits for management of a chronic condition. In the case of many CAM practitioners visited, however, a higher proportion were visited for the purpose of well-being. Respondents rated attendance at conventional and CAM practitioners equally highly. Very similar results were obtained from respondents regarding actual CAM practices obtained from providers, although this question may be complicated by the fact that certain CAM therapies can be delivered by people who do not self-identify as CAM providers (e.g. acupuncture/needling by physiotherapists, laser acupuncture by oral medicine specialists [26]. In total, 16 diverse CAM practices were used by varying degrees among 66 (22.2%) respondents, with 26 of these (39%) using multiple modalities. Multiple attendances at various CAM providers heighten concerns regarding treatment and financial burden to patients, in addition to efficacy and potential for drug interactions.

When asked about self-selected CAM product usage, 192 (64.9%) reported use of at least one such product, with a majority reporting multiple product usage. This is higher than that reported in general population studies, such as 47.7% reported in a Norwegian study [13] and higher than that reported among more specific cohorts, such as the 13.1% of present or previous cancer patients using ‘natural remedies’ (which did not however include vitamins or minerals), also in Norway [27]. Some studies have not counted vitamins as CAM products due to differences in how they are considered in different jurisdictions. We did not include disclosures of prescribed products such as high dose vitamin D and vitamin B12 injections among the figures reported herein. Among our cohort, vitamins and minerals were more commonly used than other supplements and accounted for most of the top ten individual supplements listed. The benefits of supplementary antioxidants such as vitamins A and D on tear film health have been studied, with varying results [28]. Overall, data suggest modest improvement in tear film health although reservations around optimum dosage, composition and indications remain.

In addition to vitamins and minerals, sizeable numbers (46, 15.5%) and (115, 38.9%) of respondents used various herbs and food supplements respectively, again highlighting issues of quality, safety and efficacy. The literature acknowledges that although some such products may offer promise, e.g. in supporting oral health [29], many questions remain. Some patients did not know the names of the herbal products they were taking, listing general descriptions such as ‘Chinese herbs not sure of name’ or ‘Mix specially prepared for me’. In terms of evidence-base, traditional Chinese medicine (TCM) has arguably been most evaluated among CAM approaches for Sjögren’s, although it was not commonly reported in our study. In TCM frameworks, primary Sjögren’s is characterised as “*dryness-bi*”, an intersection of congenital deficiency and external pathogenic factors, with treatment strategies attempting to counter this imbalance and support key systems, with a further emphasis on detoxification [30]. Many different TCM treatments are described; by nature, most are complex herbal mixtures, used variously in the form of pills, granules, decoctions and other extracts. In terms of studies with patients, few individual herbs have been evaluated, although peony and *Tripterygium wilfordii* glycosides, while originating from TCM, have been administered outside a classical TCM context [31]. Insufficient knowledge regarding active constituents, quality control, pharmacokinetics, toxicity and modern preparations of utilised TCM in Sjögren’s have been noted as the primary drawbacks to its acceptance and use. The two most frequently reported herbal substances in our study were turmeric and ginger, both originating in herbal traditions of the East.

Frequently reported food supplements in our cohort were probiotics and various omega-acid supplements. Of these, omega-3 and − 6 fatty acids, such as fish oils and GLA respectively, have been promoted in rheumatological illnesses, including Sjögren’s, yet products are very variable in composition and strength, and available evidence is conflicting [3, 32]. In the context of probiotic usage, autoimmune diseases including Sjögren’s have shown modifications of the microbiome as regards intestinal tract and oral flora [33], and it has been suggested that probiotics therefore offer hope as therapies. Indeed, while some preliminary human studies suggest their potential in reducing candidal colonization to prevent oral candidosis in patients with Sjögren’s [34], the majority of data stems from animal models, and uncertainties exist regarding optimal microbial strains, posology and treatment duration. In the context of side-effects, few patients reported this as an issue with CAM practices, although disease progression was once mentioned, highlighting a potential concern if CAM use replaces rather than supports conventional medicine. In the case of other practices, both traditional and laser acupuncture have been assessed in small studies involving Sjögren’s patients. Whereas traditional acupuncture had variable effects on oral [35] and ocular [36] symptoms of Sjögren’s, a pilot study examining the effects of laser acupuncture on salivary flow rates resulted in significantly higher amounts of saliva production, both after the 5-week intervention and during 6 months of follow-up [26].

A high proportion (almost 70%) of respondents utilised self-help approaches, in line with other studies [11–14]. Meditation, yoga and relaxation techniques were especially popular and primarily targeted improvement in well-being and were highly rated as helpful. There is some preliminary evidence to support their use in improving quality of life among those with Sjögren’s. A pilot study evaluated the impact of an 8-week Mindfulness-Based Stress Reduction (MBSR) program in 21 patients. Participants were trained to focus on all sensations and parts of the body using a variety of techniques. The intervention resulted in significant improvements in quality of life, degree of fatigue and discomfort levels among participants [37]. I-CAM-Q does not routinely ask about side-effects: we sought this information in our survey, which is particularly relevant given both the frequent co-morbidities and high overall use of CAM in this demographic. Higher levels of CAM use, notably of herbs and supplements, elevate the risk of clinically significant drug interactions, including elevated toxicities or treatment failures. While the use of CAM practices was not associated with either high overall levels or individually reported serious side-effects in this cohort, nonetheless it remains a valid concern and justifies the importance of screening for global use of all concomitant medicines, supplements and complementary alternative practices in those living with Sjögren’s, and should be incorporated into best-practice guidance for healthcare professionals.

### Patient perspective

It is important to recognise that many patients report finding CAM therapies beneficial. The use of CAM may allow Sjögren’s patients to take a holistic approach to their health and wellbeing, including mental health, and this same approach should be adopted by any healthcare professional that is treating or prescribing for a patient with Sjögren’s.

### Study limitations

A potential limitation of this study is that not all respondents had an official diagnosis (although a substantial majority of 88.5% did). The deliberate decision to include people awaiting a formal diagnosis was made in collaboration with our patient organisation co-authors because there are well-documented challenges with receiving an accurate and timely diagnosis, and patients experience the same symptoms during this time. We feel that it is essential to ensure the patient perspective is embedded in our research through PPI so that it addresses the most pressing needs of those affected by it. Also, a sizeable proportion reported concomitant diseases which may also affect their CAM use, but experiencing multiple diseases is typical for people with Sjögren’s or Sjögren’s symptoms, and the question specifically asked re CAM use for Sjögren’s. As the total study population reflected an international group, and we did not collect some potentially relevant data such as disease severity or respondent education attainment that may be relevant, it is not possible to establish variations in pattern based on geographical location, disease activity, or educational attainment however a high overall use is consistent, and future work will consider these variables. Challenge in identification of some of the complex CAM remedies utilised (e.g. complex herbal mixtures) and variations in how certain minerals and vitamins are considered or categorised may further complicate a full understanding. As this was an exploratory study, no target sample size was calculated, therefore the study conclusions have been considered in this context.

These findings have important implications for conventional healthcare professionals providing care to people living with Sjögren’s. Given over 80% of participants reported using one or more CAM therapies, it is important to anticipate this and ensure to proactively and openly ask people living with Sjögren’s or symptoms of Sjögren’s about CAM when taking medication histories, considering drug interactions, or managing suspected adverse drug reactions. As more CAM approaches are formally included in treatment guidelines, it is likely that use may increase further. This places an onus on healthcare professionals to ensure they have adequate knowledge of CAM approaches, access to reliable resources and evidence base, and safe and effective use in combination with conventional medicine approaches to work collaboratively with people living with Sjögren’s to optimise their overall health and care. It is also important to reflect on what gaps and limitations in our current treatment approaches result in such a high use of CAM amongst people living with Sjögren’s when compared to other diseases.

## Electronic supplementary material

Below is the link to the electronic supplementary material.

Supplementary Material 1

## Declarations

### Open data

The data for this study is not publicly available as the participants did not give written consent for their data to be shared publicly, nor was this considered for approval by the research ethics committee.
