# Supplementary material for: Use of complementary and alternative medicine (CAM) among people living with Sjögren’s: a cross-sectional survey using a modified international CAM questionnaire (I-CAM-Q)
Source: Rheumatol Int. 2025 Feb 20;45(3):55. doi: 10.1007/s00296-025-05802-w (PMC11842488; doi:10.1007/s00296-025-05802-w)
Supplement: Supplementary file 1 — Supplementary Material 1 [file 296_2025_5802_MOESM1_ESM.dotx]

**Supplementary material**

Supplementary Table S1. Survey instrument: the modified I-CAM-Q survey

**Please confirm your choice**

I consent

I do not consent

**Retention of research material in the future [choose one option]**

I give permission for the data from this survey to be stored for possible future research.

I do not give permission for the data from this survey to be stored for possible future research.

**Please indicate your age by ticking the most appropriate box below:**

18 -29 years old

30 -39 years old

40 -49 years old

50 -59 years old

60 -69 years old

70 -79 years old

80 -89 years old

Other (please specify)

**What is your gender?**

Male

Female

Non-binary

Other

Prefer not to say

**Please select the most relevant option from the list below:**

I am working full time

I am working reduced hours due to disability

I am unable to work due to disability

I am retired

I was made redundant

Other (please specify)

**Select the most appropriate option from the list below:**

I live in Connaught, Ireland

I live in Leinster, Ireland

I live in Munster, Ireland

I live in Ulster, Ireland

**If you live outside of Ireland, please type where you are living in the space below**

**Please select the most relevant option from the list below:**

I am a person with diagnosed Sjögren’s

I am a person with Sjögren’s symptoms but no diagnosis

I am a carer/ family member of a person with Sjögren’s

I am a healthcare professional

Other

**If you have been diagnosed with another condition please indicate this in the space below:**

**How long have you had a Sjögren’s diagnosis?**

< 1 year

1–5 years

6–10 years

> 10 years

Not applicable

**Visiting health care providers: Health problems may be attended to by a variety of complementary and conventional health care providers.**

**Have you seen any of the following providers in the last 12 months? Tick the box to indicate Yes to all that apply.**

Doctor (GP or other)

Physiotherapist

Chiropractor

Homeopath

Acupuncturist

Herbalist

Aromatherapist

Spiritual healer

Other (please specify)

**Please indicate the main reason you last saw the provider (Check only one for each option)**

For an acute illness/condition, one that lasted less than one month

To treat a long-term health condition (one that lasted more than one month) or its symptoms

To improve well-being

**How helpful was it for you to see this provider? (Check only one for each option)**

Very

Somewhat

Not at all

Don’t know

**Complementary treatments received from healthcare professionals. Some physicians provide complementary, as well as conventional treatments.**

**Have you received any on the following complementary treatments from a health care professional in the last 12 months? Tick the box to indicate Yes to all that apply.**

Manipulation

Homeopathy

Acupuncture

Herbs

Aromatherapy

Spiritual healing

Other (please specify)

**Please indicate the number of times you received this treatment in the last 3 months**

**Please indicate the main reason you last received this treatment. (Check only one).**

For an acute illness/condition, one that lasted less than one month

To treat a long-term health condition (one that lasted more than one month) or its symptoms

To improve well-being

**How helpful was it to receive treatment? (Check only one)**

Very

Somewhat

Not at all

Don’t know

**Use of Herbal Medicine and Dietary Supplements, including tablets, capsules and liquids.**

**In the text boxes below please list the NAMES of up to 3 products you have used in the last 12 months**

Herbs/Herbal medicines

Vitamins/Minerals

Homeopathic remedies

Dietary supplements or Health Foods

**Please indicate the main reason that applies to your last use. (Check only one)**

For an acute illness/condition, one that lasted less than one month

To treat a long-term health condition (one that lasted more than one month) or its symptoms

To improve well-being

**How helpful did you find this product? (Check only one)**

Very

Somewhat

Not at all

Don’t know

**Self Help Practices**

**Have you used any of the following self help practices in the last 12 months? Tick the box to indicate Yes to all that apply.**

Meditation

Yoga

Qigong

Tai Chi

Relaxation techniques

Visualization

Aromatherapy

Prayer

Other (please specify)

**Please indicate the number of times you used this practice in the last 3 months**

**Please indicate the main reason that applies to your last use of the self-help practice.(Check only one).**

For an acute illness/condition, one that lasted less than one month

To treat a long-term health condition (one that lasted more than one month) or its symptoms

To improve well-being

**How helpful did you find this self-help practice? (Check only one)**

Very

Somewhat

Not at all

Don’t know

**Have you experienced adverse events from any of the medicines or treatments listed below in the last 12 months? Tick the box to indicate Yes to all that apply.**

Conventional medicine or treatment

Manipulation

Chiropractor

Homeopathy

Acupuncture

Aromatherapy

Herbs/Herbal medicine

Homeopathic remedies

Dietary supplements or Health Foods

Vitamins/minerals

Spiritual healing

Other (please specify)

**Please provide details on the adverse event(s) you experienced**

Supplementary Table S2. Attendance at health care providers stratified by location and age group

|  | **Frequency** | | **Reason - Outside Ireland** | | | **Reason - Ireland** | | | **Helpfulness (Very/Some)** | |
| --- | --- | --- | --- | --- | --- | --- | --- | --- | --- | --- |
|  | Outside Ireland | Ireland | Acute illness | Chronic illness | Well-being | Acute illness | Chronic illness | Well-being | Outside Ireland | Ireland |
| Doctor (GP or other) | 112 (84.9%) | 151 (92.1%) | 21 (18.8%) | 14 (12.5%) | 72 (64.3%) | 40 (26.5%) | 11 (7.3%) | 93 (61.6%) | 98 (87.5%) | 135 (89.4%) |
| Physiotherapist | 41 (31.1%) | 63 (38.4%) | 10 (24.4%) | 5 (12.2%) | 26 (63.4%) | 16 (25.4%) | 8 (12.7%) | 36 (57.1%) | 37 (90.2%) | 57 (90.5%) |
| Chiropractor | 17 (12.9%)* | 8 (4.9%) | 4 (23.5%) | 2 (11.8%) | 8 (47.1%) | 1 (12.5%) | 0 (0.0%) | 6 (75.0%) | 14 (82.4%) | 6 (75.0%) |
| Homeopath | 5 (3.8%) | 7 (4.3%) | 0 (0.0%) | 3 (60.0%) | 2 (40.0%) | 0 (0.0%) | 1 (14.3%) | 6 (85.7%) | 4 (80.0%) | 5 (71.4%) |
| Acupuncturist | 7 (5.3%) | 16 (9.8%) | 2 (28.6%) | 0 (0.0%) | 5 (71.4%) | 0 (0.0%) | 2 (12.5%) | 14 (87.5%) | 5 (71.4%) | 12 (75.0%) |
| Herbalist | 5 (3.8%) | 7 (4.3%) | 0 (0.0%) | 2 (40.0%) | 3 (60.0%) | 0 (0.0%) | 5 (71.4%) | 2 (28.6%) | 3 (60.0%) | 6 (85.7%) |
| Aromatherapist | 2 (1.5%) | 3 (1.8%) | 0 (0.0%) | 1 (50.0%) | 1 (50.0%) | 0 (0.0%) | 3 (100.0%) | 0 (0.0%) | 2 (100.0%) | 3 (100.0%) |
| Spiritual healer | 2 (1.5%) | 5 (3.1%) | 0 (0.0%) | 2 (100.0%) | 0 (0.0%) | 0 (0.0%) | 4 (80.0%) | 1 (20.0%) | 2 (100.0%) | 4 (80.0%) |
| Other CAM practitioner | 9 (6.8%) | 11 (6.7%) | 0 (0.0%) | 5 (55.6%) | 4 (44.4%) | 1 (9.1%) | 4 (36.4%) | 5 (45.5%) | 8 (88.9%) | 10 (90.9%) |
| Any CAM practitioner | 34 (25.8%) | 39 (23.8%) |  |  |  |  |  |  |  |  |
|  | Frequency | | Reason - Age <60 | | | Reason - Age ≥60 | | | Helpfulness (Very/Some) | |
|  | Age <60 | Age ≥60 | Acute illness | Chronic illness | Well-being | Acute illness | Chronic illness | Well-being | Age <60 | Age ≥60 |
| Doctor (GP or other) | 157 (88.7%) | 106 (89.1%) | 35 (22.3%) | 14 (8.9%) | 103 (65.6%) | 26 (24.5%) | 11 (10.4%) | 62 (58.5%) | 133 (84.7%) | 100 (94.3%)* |
| Physiotherapist | 54 (30.5%) | 50 (42.0%)* | 13 (24.1%) | 8 (14.8%) | 31 (57.4%) | 13 (26.0%) | 5 (10.0%) | 31 (62.0%) | 49 (90.7%) | 45 (90.0%) |
| Chiropractor | 13 (7.3%) | 12 (10.1%) | 2 (15.4%) | 1 (7.7%) | 8 (61.5%) | 3 (25.0%) | 1 (8.3%) | 6 (50.0%) | 12 (92.3%) | 8 (66.7%) |
| Homeopath | 8 (4.5%) | 4 (3.4%) | 0 (0.0%) | 3 (37.5%) | 5 (62.5%) | 0 (0.0%) | 1 (25.0%) | 3 (75.0%) | 6 (75.0%) | 3 (75.0%) |
| Acupuncturist | 14 (7.9%) | 9 (7.6%) | 0 (0.0%) | 1 (7.1%) | 13 (92.9%) | 2 (22.2%) | 1 (11.1%) | 6 (66.7%) | 11 (78.6%) | 6 (66.7%) |
| Herbalist | 7 (4.0%) | 5 (4.2%) | 0 (0.0%) | 3 (42.9%) | 4 (57.1%) | 0 (0.0%) | 4 (80.0%) | 1 (20.0%) | 4 (57.1%) | 5 (100.0%) |
| Aromatherapist | 3 (1.7%) | 2 (1.7%) | 0 (0.0%) | 2 (66.7%) | 1 (33.3%) | 0 (0.0%) | 2 (100.0%) | 0 (0.0%) | 3 (100.0%) | 2 (100.0%) |
| Spiritual healer | 6 (3.4%) | 1 (0.8%) | 0 (0.0%) | 5 (83.3%) | 1 (16.7%) | 0 (0.0%) | 1 (100.0%) | 0 (0.0%) | 5 (83.3%) | 1 (100.0%) |
| Other CAM practitioner | 165 (93.2%) | 111 (93.3%) | 1 (8.3%) | 5 (41.7%) | 5 (41.7%) | 0 (0.0%) | 4 (50.0%) | 4 (50.0%) | 11 (91.7%) | 7 (87.5%) |
| Any CAM practitioner | 43 (24.3%) | 30 (25.2%) |  |  |  |  |  |  |  |  |

* Chi squared test p<0.05

Supplementary Table S3. CAM treatments obtained from providers stratified by location and age group

|  | **Frequency** | | **Reason - Outside Ireland** | | | **Reason - Ireland** | | | **Helpfulness (Very/Some)** | |
| --- | --- | --- | --- | --- | --- | --- | --- | --- | --- | --- |
|  | Outside Ireland | Ireland | Acute illness | Chronic illness | Well-being | Acute illness | Chronic illness | Well-being | Outside Ireland | Ireland |
| Manipulation | 13 (9.9%) | 14 (8.5%) | 1 (7.7%) | 2 (15.4%) | 10 (76.9%) | 2 (14.3%) | 2 (14.3%) | 10 (71.4%) | 12 (92.3%) | 14 (100.0%) |
| Homeopathy | 5 (3.8%) | 7 (4.3%) | 0 (0.0%) | 1 (20.0%) | 4 (80.0%) | 0 (0.0%) | 0 (0.0%) | 7 (100.0%) | 4 (80.0%) | 5 (71.4%) |
| Acupuncture | 8 (6.1%) | 17 (10.4%) | 4 (50.0%) | 0 (0.0%) | 4 (50.0%) | 0 (0.0%) | 1 (5.9%) | 15 (88.2%)* | 7 (87.5%) | 14 (82.4%) |
| Herbs | 6 (4.6%) | 7 (4.3%) | 0 (0.0%) | 3 (50.0%) | 3 (50.0%) | 0 (0.0%) | 2 (28.6%) | 5 (71.4%) | 5 (83.3%) | 6 (85.7%) |
| Aromatherapy | 0 (0.0%) | 1 (0.6%) | 0 (0.0%) | 0 (0.0%) | 0 (0.0%) | 0 (0.0%) | 1 (100.0%) | 0 (0.0%) | 0 (0.0%) | 1 (100.0%) |
| Spiritual healing | 1 (0.8%) | 6 (3.7%) | 0 (0.0%) | 1 (100.0%) | 0 (0.0%) | 0 (0.0%) | 5 (83.3%) | 1 (16.7%) | 1 (100.0%) | 6 (100.0%) |
|  | Frequency | | Reason - Age <60 | | | Reason - Age ≥60 | | | Helpfulness (Very/Some) | |
|  | Age <60 | Age ≥60 | Acute illness | Chronic illness | Well-being | Acute illness | Chronic illness | Well-being | Age <60 | Age ≥60 |
| Manipulation | 13 (7.3%) | 14 (11.8%) | 0 (0.0%) | 2 (15.4%) | 11 (84.6%) | 3 (21.4%) | 2 (14.3%) | 9 (64.3%) | 13 (100.0%) | 13 (92.9%) |
| Homeopathy | 8 (4.5%) | 4 (3.4%) | 0 (0.0%) | 1 (12.5%) | 7 (87.5%) | 0 (0.0%) | 0 (0.0%) | 4 (100.0%) | 6 (75.0%) | 3 (75.0%) |
| Acupuncture | 14 (7.9%) | 11 (9.2%) | 2 (14.3%) | 0 (0.0%) | 12 (85.7%) | 2 (18.2%) | 1 (9.1%) | 7 (63.6%) | 13 (92.9%) | 8 (72.7%) |
| Herbs | 9 (5.1%) | 4 (3.4%) | 0 (0.0%) | 3 (33.3%) | 6 (66.7%) | 0 (0.0%) | 2 (50.0%) | 2 (50.0%) | 7 (77.8%) | 4 (100.0%) |
| Aromatherapy | 0 (0.0%) | 1 (0.8%) | 0 (0.0%) | 0 (0.0%) | 0 (0.0%) | 0 (0.0%) | 1 (100.0%) | 0 (0.0%) | 0 (0.0%) | 1 (100.0%) |
| Spiritual healing | 5 (2.8%) | 2 (1.7%) | 0 (0.0%) | 4 (80.0%) | 1 (20.0%) | 0 (0.0%) | 2 (100.0%) | 0 (0.0%) | 5 (100.0%) | 2 (100.0%) |

* Fisher’s exact test p<0.05

Supplementary Table S4. Use of self-help practices stratified by location and age group

|  | **Frequency** | | **Reason - Outside Ireland** | | | **Reason - Ireland** | | | **Helpfulness (Very/Some)** | |
| --- | --- | --- | --- | --- | --- | --- | --- | --- | --- | --- |
|  | Outside Ireland | Ireland | Acute illness | Chronic illness | Well-being | Acute illness | Chronic illness | Well-being | Outside Ireland | Ireland |
| Meditation | 38 (28.8%) | 50 (30.5%) | 1 (2.6%) | 29 (76.3%) | 7 (18.4%) | 1 (2.0%) | 32 (64.0%) | 15 (30.0%) | 35 (92.1%) | 44 (88.0%) |
| Yoga | 28 (21.2%) | 47 (28.7%) | 1 (3.6%) | 18 (64.3%) | 9 (32.1%) | 0 (0.0%) | 30 (63.8%) | 16 (34.0%) | 22 (78.6%) | 43 (91.5%) |
| Qigong | 9 (6.8%)* | 2 (1.2%) | 0 (0.0%) | 7 (77.8%) | 1 (11.1%) | 0 (0.0%) | 0 (0.0%) | 2 (100.0%) | 8 (88.9%) | 2 (100.0%) |
| Taichi | 6 (4.6%) | 8 (4.9%) | 0 (0.0%) | 4 (66.7%) | 2 (33.3%) | 0 (0.0%) | 4 (50.0%) | 4 (50.0%) | 1 (16.7%) | 7 (87.5%)* |
| Relaxation techniques | 45 (34.1%) | 47 (28.7%) | 2 (4.4%) | 25 (55.6%) | 16 (35.6%) | 0 (0.0%) | 28 (59.6%) | 18 (38.3%) | 42 (93.3%) | 45 (95.7%) |
| Visualisation | 8 (6.1%) | 18 (11.0%) | 0 (0.0%) | 5 (62.5%) | 3 (37.5%) | 0 (0.0%) | 11 (61.1%) | 6 (33.3%) | 8 (100.0%) | 14 (77.8%) |
| Aromatherapy | 11 (8.3%) | 12 (7.3%) | 0 (0.0%) | 6 (54.6%) | 5 (45.5%) | 1 (8.3%) | 6 (50.0%) | 4 (33.3%) | 10 (90.9%) | 10 (83.3%) |
| Prayer | 29 (22.0%) | 37 (22.6%) | 0 (0.0%) | 25 (86.2%) | 2 (6.9%) | 0 (0.0%) | 28 (75.7%) | 7 (18.9%) | 24 (82.8%) | 32 (86.5%) |
|  | Frequency | | Reason - Age <60 | | | Reason - Age ≥60 | | | Helpfulness (Very/Some) | |
|  | Age <60 | Age ≥60 | Acute illness | Chronic illness | Well-being | Acute illness | Chronic illness | Well-being | Age <60 | Age ≥60 |
| Meditation | 52 (29.4%) | 36 (30.3%) | 1 (1.9%) | 34 (65.4%) | 15 (28.9%) | 1 (2.8%) | 27 (75.0%) | 7 (19.4%) | 46 (88.5%) | 33 (91.7%) |
| Yoga | 49 (27.7%) | 26 (21.9%) | 1 (2.0%) | 31 (63.3%) | 17 (34.7%) | 0 (0.0%) | 17 (65.4%) | 8 (30.8%) | 42 (85.7%) | 23 (88.5%) |
| Qigong | 5 (2.8%) | 6 (5.0%) | 0 (0.0%) | 3 (60.0%) | 2 (40.0%) | 0 (0.0%) | 4 (66.7%) | 1 (16.7%) | 4 (80.0%) | 6 (100.0%) |
| Taichi | 5 (2.8%) | 9 (7.6%) | 0 (0.0%) | 2 (40.0%) | 3 (60.0%) | 0 (0.0%) | 6 (66.7%) | 3 (33.3%) | 4 (80.0%) | 4 (44.4%) |
| Relaxation techniques | 55 (31.1%) | 37 (31.1%) | 2 (3.6%) | 26 (47.3%) | 25 (45.5%) | 0 (0.0%) | 27 (73.0%) | 9 (24.3%) | 52 (94.6%) | 35 (94.6%) |
| Visualisation | 14 (7.9%) | 12 (10.1%) | 0 (0.0%) | 8 (57.1%) | 5 (35.7%) | 0 (0.0%) | 8 (66.7%) | 4 (33.3%) | 11 (78.6%) | 11 (91.7%) |
| Aromatherapy | 15 (8.5%) | 8 (6.7%) | 1 (6.7%) | 6 (40.0%) | 7 (46.7%) | 0 (0.0%) | 6 (75.0%) | 2 (25.0%) | 12 (80.0%) | 8 (100.0%) |
| Prayer | 37 (20.9%) | 29 (24.4%) | 0 (0.0%) | 29 (78.4%) | 6 (16.2%) | 0 (0.0%) | 24 (82.8%) | 3 (10.3%) | 33 (89.2%) | 23 (79.3%) |

* Fisher’s exact test p<0.05
